# Exploring lifestyle and risk in preventing type 2 diabetes-a nested qualitative study of older participants in a lifestyle intervention program (VEND-RISK)

**DOI:** 10.1186/s12889-016-3559-y

**Published:** 2016-08-25

**Authors:** Ingrid S. Følling, Marit Solbjør, Kristian Midthjell, Bård Kulseng, Anne-S Helvik

**Affiliations:** 1 Department of Health Sciences, Nord University, Røstad, N-7600 Levanger, Norway; 2 Department of Public Health and General Practice, Faculty of Medicine, Norwegian University of Science and Technology, Postboks 8905, MTFS, 7491 Trondheim, Norway; 3 St. Olavs University Hospital, Trondheim, Norway; 4 Department of Community Health and General Practice, HUNT Research Centre, Faculty of Medicine, Norwegian University of Science and Technology, Trondheim, Norway; 5 Obesity Research Centre, St. Olavs University Hospital, Forsyningssenteret, 7006 Trondheim, Norway; 6 Department of Cancer Research and Molecular Medicine, Faculty of Medicine, Norwegian University of Science and Technology, Postboks 8905, N-7491 Trondheim, Norway; 7 Norwegian National Advisory Unit for Aging and Health, Vestfold Health Trust, Tønsberg, 3103 Norway; 8 Department of Social Work and Health Science, Faculty of Social Sciences and Technology Management, Norwegian University of Science and Technology, 7491 Trondheim, Norway

**Keywords:** Type 2 diabetes risk, Lifestyle, Lifestyle intervention, Salutogenesis, The HUNT study, Qualitative research, Semi-structured interviews

## Abstract

**Background:**

Lifestyle intervention may reduce the development of type 2 diabetes among high-risk individuals. The aim of this study was to explore how older adults perceived their own lifestyle and being at increased risk for type 2 diabetes while they participated in a lifestyle intervention programme.

**Methods:**

A nested qualitative study was performed with 26 participants (mean age 68 years) in the VEND-RISK Study. Participants had previously participated in the HUNT3 Study and the HUNT DE-PLAN Study, where their risk for developing type 2 diabetes (FIND-RISC ≥ 15) had been identified. The data were analysed using systematic text condensation.

**Results:**

Two main themes were identified. The first theme was having resources available for an active lifestyle, which included having a family and being part of a social network, having a positive attitude toward life, and maintaining established habits from childhood to the present. The second theme was being at increased risk for type 2 diabetes, which included varied reactions to the information on increased risk, how lifestyle intervention raised awareness about risk behaviour, and health-related worries and ambitions as type 2 diabetes prevention.

**Conclusions:**

Assessing a participant’s resources could improve the outcomes of lifestyle intervention programmes. Both family history and risk perception could be used in preventive strategies to enhance changes in lifestyle.

**Trial registration:**

The VEND-RISK Study was registered in ClinicalTrials.gov on April 26, 2010, with the registration number NCT01135901.

## Background

Type 2 diabetes has increased rapidly over the last thirty years, as has prediabetes in middle-aged and older adults [1]. Of all diseases measured in years lived with disability, type 2 diabetes has increased the most from 1990 to 2013 [2].

Several studies have shown that type 2 diabetes can be prevented when individuals at increased risk make lifestyle changes [3–6], even with modest clinical efforts [5]. The World Health Organization has estimated that 90 % of type 2 diabetes can be prevented through changes in diet, physical activity and smoking habits [7]. In order to prevent type 2 diabetes, it is important to develop tools and strategies to help individuals at high risk to make lifestyle changes [8].

A healthy lifestyle is associated with keeping risk factors at low levels [9]. Intervention programmes for healthier lifestyle offered by primary health care services have been found to be feasible and effective for individuals at high risk for type 2 diabetes [10]. During the last ten years, health authorities in Norway have recommended municipalities to establish services for people with unhealthy lifestyles, highlighting the need for preventing type 2 diabetes [11]. Lifestyle intervention programmes in municipalities are recommended to be based on a salutogenic theoretical approach [12]. The main essence of the salutogenic theory is sense of coherence, which refers to the ability to use one’s own resources [13], including the ability to understand what is happening, the ability to manage the situation alone or with the help of significant others, and to find meaning in the situation [14]. A high sense of coherence is associated with better future health [14, 15]. In individuals at risk for type 2 diabetes, a high sense of coherence is found to be associated with lifestyle change [16]. However, such associations are contested. A study that included individuals aged 50 years or older at increased risk for type 2 diabetes found no association between a high sense of coherence and developing type 2 diabetes [17].

How information about risk is processed and understood may depend on social and psychological factors, including both family history and present lifestyle [18]. Several quantitative studies have elaborated on type 2 diabetes prevention [3–6, 10] and risk perception in relation to lifestyle [19–22]. One study found that individuals at high risk for type 2 diabetes did not have a higher awareness about the importance of diet and weight management as a means to prevent type 2 diabetes than those without risk [19]. Another study found that a higher age of those at risk was associated with lesser expectations and lower readiness for lifestyle change [20]. However, interventions that involved and changed risk perceptions successfully, regardless of age, seemed to change towards a healthier behaviour [21, 22].

Despite numerous quantitative studies on type 2 diabetes, lifestyle and risk [3–6, 10, 19–22], there is still a need for qualitative studies on these issues. Qualitative studies are well suited to explore and provide rich descriptions of complex phenomena [23]. Previous studies have explored perceptions of risk for type 2 diabetes and lifestyle change from individuals at increased risk [24–26]. These studies have found that people with prediabetes were surprised about their risk [24, 25]. A study of participants’ experiences with screening for type 2 diabetes found that those who were at high risk were relieved to see that they were only at risk, but had no intention to change their lifestyle [26]. Perceptions of risk for type 2 diabetes may change during a stepwise method developed to help individuals adapt psychologically to their risk [25]. However, little is known about how those at increased risk for type 2 diabetes understand their lifestyle and how they perceive their risk when they choose to attend a lifestyle intervention programme. Thus, the aim of this study was to explore how older adults who are participants in a lifestyle intervention programme experience their own lifestyle and being at increased risk for type 2 diabetes.

## Methods

This study used a nested qualitative approach with individual semi-structured in-depth interviews. The design enabled us to obtain rich data about informants’ perceptions about their lifestyle and being at increased risk for type 2 diabetes, contextualized in the setting of attending a lifestyle intervention programme (the VEND-RISK Study). As a theoretical framework for our explorative analysis, we used the main concept sense of coherence from the salutogenic theory [14, 15].

### Sample size and recruitment

The sample selection goes retrospectively back through three studies: the Nord-Trøndelag Health Study 3 (HUNT3), the HUNT DE-PLAN Study and the VEND-RISK Study.

The HUNT Study is a large population study in the county of Nord-Trøndelag in Norway with three surveys over the last 30 years [27]. The third HUNT Study in 2006-08 identified about 5000 people as being at a high risk (>30 %) of developing type 2 diabetes over the next ten years, based on the Finnish Diabetes Risk Score (FIND-RISC) questionnaire. The questionnaire includes eight questions about traditional risk factors for type 2 diabetes and is considered the best screening tool to use in a Caucasian population [28, 29]. A FIND-RISC score of 15 or more with an index from 0–26 means having at least 30 % increased risk of developing type 2 diabetes during the upcoming ten years [30]. Individuals without known diabetes and a score of >15 received oral and written notices about their risk.

Furthermore, these individuals identified at increased risk for type 2 diabetes were eligible participants for the HUNT arm of an international multicentre study, the DE-PLAN Study (Diabetes in Europe. Prevention through Lifestyle, physical Activity and Nutrition). They received a letter and a phone call from a study nurse inviting them to attend the DE-PLAN Study [31]. The first step of the DE-PLAN Study was an oral glucose tolerance test, identifying some people who had already contracted diabetes. These were referred to their own physician for diagnostic follow-up and treatment, and they were not invited to further follow-up in DE-PLAN. The DE-PLAN Study offered participants the opportunity to attend informational meetings including the importance of avoiding type 2 diabetes, and how this could be prevented through simple nutritional advice. Also, the meetings addressed how to be more active and participants were informed about physical activities in their local communities. Furthermore, it was recommended that the individuals avoid gaining weight.

**Fig. 1:**
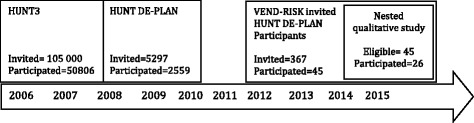
Timeline showing the sample selection from the HUNT3 Study, the HUNT DE-PLAN Study and the HUNT DE PLAN participants who were involved in the VEND-RISK Study, which were eligible informants for the present study

In 2012, the VEND-RISK Study was initiated in two of the municipalities were the HUNT DE-PLAN had taken place. All participants who had been involved in the HUNT DE-PLAN (*n* = 322) received an information letter about participation in the VEND-RISK Study. Eligible participants’ names and addresses were retrieved from the HUNT DE-PLAN participant list for these two municipalities. The VEND-RISK Study included a more intensive lifestyle intervention programme provided by the primary health care services in local municipalities. VEND-RISK was designed for overweight people at increased risk for type 2 diabetes, with the goal of stimulating participants to be more physically active and to eat a healthier diet. VEND-RISK offered various physical activities lead by physiotherapists twice a week, and nutritional courses with a nutritionist. In addition, information meetings with themes relevant for type 2 diabetes risk were held once a year. The study also included annual surveys, blood sample testing and physical activity tests for five years. Altogether 45 out of 322 DE-PLAN participants from the two municipalities agreed to be involved in the VEND-RISK Study, and were eligible for selection to participate at interviews in this present study. Figure 1 illustrates the timeline for the sampling process for participants included in the present nested qualitative study.

One nurse working with the VEND-RISK Study helped in the recruiting process. The nurse phoned potential participants, starting at the top of the alphabetical list of the 45 individuals eligible for the study. Appointments were made one week before each interview. During the phone-call, the nurse informed participants about the qualitative study and everybody who were invited accepted to be interviewed. Recruitment proceeded continuously until 26 participants had been interviewed, at which point the interviews brought no new information. All interviews were conducted during a period of six weeks. The 26 participants that were interviewed were similar regarding age and gender for all 45 eligible participants. The 19 participants not being interviewed continued the lifestyle program in the VEND-RISK Study as planned, together with the 26 participants that were interviewed.

### Informants

Twenty-six ethnic Norwegian informants aged 59–75 years (mean age 68) were interviewed. The informants’ health measurements at VEND-RISK baseline inclusion showed a mean body mass index (BMI) of 30.2 (Standard Deviation (SD) 3.4) with a mean FIND-RISC score of 17 (SD 3.2). Sociodemographic variables and health measurements of informants are described in Table 1.

**Table 1:** Informants characteristics

Characteristics	Total (*N* = 26)
Sociodemographic variables	*N* (%)
Gender	
Female	15 (58)
Male	11 (42)
Age	
59–64	4 (16)
65–69	17 (65)
≥ 70	5 (19)
Civil status	
Partner/married	20 (77)
Divorced/widowed	6 (23)
Highest level of education	
Nine years or less of school	5 (19)
More than nine years of school	14 (54)
Bachelor degree or higher	7 (27)
Work status	
Disability leave	6 (23)
Partly retired (1–49 %)	4 (16)
Working 50 % or more	4 (16)
Retired (100 %)	12 (46)
Essential health measures	
Family history of type 2 diabetes	
Present	17 (65)
Not present	9 (35)
Weight Categories^a^	
Normal weight (BMI 18,5–24,9 kg/m^2^)	1 (4)
Overweight (BMI 25–29,9 kg/m^2^)	11 (42)
Obese (BMI ≥30 kg/m^2^)	14 (54)
Waist Circumference (cm)	Mean (SD)
Men	107.5 (6.4)
Women	103.5 (10.1)
HbA_1c_ (mmol/mol)	5.8 (0.5)

^a^Weight category definitions are based on the World Health Organizations (WHO) Body Mass Index

(BMI) cutoffs

### Interviews and interview guide

Individual in-depth interviews were conducted over six weeks in spring 2015 at a local outpatient care facility that served the two municipalities. Interviews lasted between 15 and 73 min (mean duration 28 min). The first author conducted all interviews. Additional notes and reflections were written down immediately after each interview.

The interview guide was semi-structured with open-ended questions, allowing informants to speak freely about what they considered essential to their lifestyle and being at increased risk. Main interview questions were: “*How has your health and lifestyle been through your life?*”, “*How did you react to the information about being at risk for type 2 diabetes?”* and *“How has the VEND-RISK intervention programme influenced your lifestyle?”* . The interviews proceeded as a conversation, with follow-up questions *“Did you do any changes based on the knowledge about your risk?” and “What experience do you have with changing habits in diet and exercise?”*, with the goal of exploring what informants considered to be important.

### Data analysis

Audio recordings of all 26 interviews were transcribed verbatim. Systematic text condensation based on a phenomenological approach were used in the analysis [32, 33]. In the first step of the analysis, the first author read all transcribed interviews and interview notes to get an overall impression. A mind map was made for preliminary themes that were identified during the first reading. In the second step, all meaning units derived from the material were sorted into codes. Codes were compared and categorized into main themes and subthemes. In the third step, themes and codes were summarized, read and discussed, with the goal of finding the essence in the material that reflected the participants’ narratives. The second and last author read three interviews and a summary of the interviews and met to discuss codes and themes with the first author. After several meetings, codes and themes were adjusted and renamed, and the content of themes and subthemes was condensed. In the end, all findings were summarized and concepts in the themes and subthemes were grouped. The findings were continuously checked against the transcription for validation during the analysis and discussions of themes. NVivo 10.0 was used as a systematization tool.

All quotes presented in the results section are translated from Norwegian to English and anonymized.

## Results

**Fig. 2:**
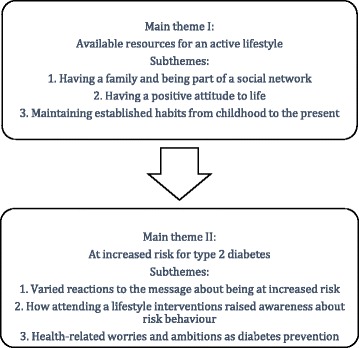
Main themes and subthemes

The study revealed two main themes with three subthemes each (see Fig. 2). The first main theme was “Available resources for an active lifestyle”. Subthemes were “Having a family and being part of a social network”, “Having a positive attitude to life”, and “Maintaining established habits from childhood to the present”. The second main theme was “Being at increased risk for type 2 diabetes”. The subthemes were “Varied reactions to the message about being at risk”, “How lifestyle intervention (VEND-RISK) raised awareness about risk behaviour”, and “Health-related worries and ambitions as type 2 diabetes prevention”.

In the stories, the first main theme was presented as a foundation that affected the second main theme.

### Main theme I: available resources for active lifestyle

#### Having a family and being part of a social network

In the informants’ narratives, having a family with children, grandchildren and siblings nearby was essential for an active lifestyle. In addition, being part of a social network was seen as important for their activity habits. They highlighted being engaged in organizations and local community groups, exemplified by political engagements, senior groups, company sport groups and voluntary work. The social aspect of being occupied with different activities was emphasized in their stories, as typified by one informant:

*I attend the health association every Monday and the knitting club every Tuesday. I also go bowling once a week. I have to go out to see people so this social aspect is most important for me. Woman, age 63*

#### Having a positive attitude to life

Informants accentuated that they had a positive attitude to life. They saw themselves as responsible for their own happiness and quality of life. They asserted that it was important to be engaged in life and activities, and emphasized that there was nothing to be gained by feeling sorry for themselves. They were grateful for what life had given them. In spite of traumatic episodes and negative health conditions, they said that they would not let sadness ruin their lives. It became apparent that they turned to their families and social networks to get through difficult life experiences. To acknowledge their resources, to be able to use them and to have a positive attitude was essential for staying active, in spite of life difficulties. One participant said:

*I lost my wife a year ago. I had no desire to sit thinking about this. I have a positive attitude. I go travelling with friends, go to concerts and go out dancing… I cannot just sit at home and feel sorry for myself that is not beneficial. Man, age 66*

#### Maintaining established habits from childhood to the present

According to the informants’ narratives, they had formed habits during their childhood through an active lifestyle and healthy eating. They told that they as children, after a normal school day, helped out at home or played outside. They walked or bicycled to get around, a habit that continued through their adult life. As described in their stories about childhood eating habits, food was made with natural ingredients. In the views of the study participants, food habits from their upbringing continued into adulthood, and they told that they still considered regular meals and a low sugar intake as important for a healthy lifestyle.

Not many had been involved in organized sports as children and adolescents, but they were used to being out hiking, skiing, ice skating and walking in the woods. When talking about exercise in their adult life, some informants described going to fitness centres, while others attended organized sport activities. Participants said it was common for them to go for walks with their family on the weekends. They perceived the recommendation for 30 min of daily physical activity as healthy behaviour for maintaining an active lifestyle.

Being active was important for their quality of life. However, their stories described more than just physical activity. Activities involving family and friends were highlighted as important, whether going to a concert or the swimming pool. In addition, they described everyday activities such as housework and garden work as important. One said:

*I am active with daily activities. I go skiing in the winter, I shovel snow, I chop wood and I have a house to keep clean. I pick berries in the woods. I cook for my family and I have my loom, so I do not sit in a chair. Woman, age 67*

### Main theme II: at increased risk for type 2 diabetes

#### Varied reactions to the information about being at increased risk

The informants’ stories varied along a continuum in terms of the consequences the risk information had on their lifestyle. They felt that receiving the information about being at increased risk for type 2 diabetes from the HUNT3 study was valuable. Some informants remembered being told that their established food habits and the fact that they already met activity recommendations were good strategies for continuing their lives without needing to be worried about their increased risk. Others were surprised about being at increased risk and talked about having reduced their sugar intake after they were given this information. However, most informants thought little about making lifestyle changes because of the risk information provided by the HUNT3 survey (see Table 2). They described themselves as being active individuals, which made their increased type 2 diabetes risk less of a concern. Informants recalled very little about attending the HUNT DE-PLAN Study.

**Table 2:** Informants’ remarks regarding the second main theme, an increased risk for type 2 diabetes

Varied reactions to the information about being at increased risk (HUNT 3):
*“It was okay to get the information and to learn about my health status.” Man, age 66*
*“It was good to get the message, but I can’t go around being afraid of getting type 2 diabetes.” Woman, age 59*
*“I stopped wanting to eat sweets when I learned I was at risk for type 2 diabetes.” Woman, age 65*
*“Actually, I thought it was strange that I was at risk, but I am glad that I found out about it.” Woman, age 65*
How attending a lifestyle intervention (the VEND-RISK Study) raised awareness about risk behaviour:
*“Now I have the chance to start prolonging my life and improving my health.” Man, age 65*
*“I thought, now I have to start; I walked before but now I walk at least two hours a day.” Woman, age 74*
*“I want to learn more about eating healthier to prevent further disease and I want to lose some weight.” Woman, age 63*
*“I have become far more conscious about the importance of activity and diet.” Man, age 65*
*“I think it’s easier to stay on track and focus on good habits with the VEND-RISK tests follow-up. It is important to get some objective feedback. It would be much easier to slip back if we did not have the measurements.” Man, age 64*
*“It is like a carrot to have the VEND-RISK study measurements.” Man, age 63*
Health related worries and ambitions as diabetes prevention:
*“My husband has diabetes, and he has had to have both legs amputated, and that makes me think about my lifestyle.” Woman, age 66*
*“My mother went blind because of diabetes, and died young. At that time there was not much known about preventing diabetes.” Man, age 67*
*“My mother had diabetes and she was obese. She struggled with pain in her joints and just sat in a chair. Because of that I try to take precautions, I ate a lot of sweets before, but now I think more about what I eat and the consequences.” Woman, age 65*
*“After my father got type 2 diabetes I’ve been very aware of my risk … Also, one of my aunts had to have both legs amputated and that scared me.” Man, age 63*
*“I don’t want to take medication, so when I learned I was at risk for diabetes I just had to do something and focus more on diet and exercise.” Woman, age 67*
*“I want to improve my health to stay well as long as possible.” Man, age 66*
*“It is not too late to do something about your diet and exercise habits; I do not want to sit on a couch and watch television for the rest of my life.” Man, age 65*

#### How attending a lifestyle intervention programme (VEND-RISK) raised awareness about risk behaviour

The informants considered the invitation to the lifestyle intervention programme (VEND-RISK) to be a reminder of their increased risk. They focused on a healthier behaviour after being included in VEND-RISK, but to varying degrees. Some had started at fitness centres and others were more aware of their diet. One informant had been to a diabetes training programme at the local hospital to learn more about type 2 diabetes. For those who participated in physical activity, established self-initiated activities were preferred over the activity interventions provided by VEND-RISK. However, even if there was variation in awareness about risk and in the efforts made by informants to have a healthier lifestyle, the stories they told showed an increased focus on being more active. Furthermore, they described that results from blood samples and physical measurements at the annual VEND-RISK tests as motivation for being more active. They also expressed the feeling that they had a social responsibility to contribute to research (see comments in Table 2).

#### Health-related worries and ambitions as type 2 diabetes prevention

Informants with diabetes in the family were worried about getting the disease themselves. Those who had family members with complications from diabetes such as nephropathy and retinopathy were more anxious. Some had obese parents with diabetes, some of whom also had obesity-related pain and reductions in mobility. The prospect of finding themselves in similar situations led to the desire to prevent the disease. They emphasized that they did not want to end up like their parents or relatives. Also, some informants mentioned medicines, needles and injections as being so frightening that they had shifted to a healthier lifestyle to avoid this kind of future for themselves. The focus on preventing type 2 diabetes had been strengthened from the time when they were given the first information on risk to the present, in part because they had grown older.

They emphasized their ambitions to stay healthy as long as possible in their stories. A family history of diabetes and ambitions of having a healthy life seemed to have had a greater influence on informants in terms of determining whether or not they had a healthier lifestyle than information about their risk alone. The informants also contradicted themselves, describing themselves as active on the one hand, while also saying that diabetes-related worries helped motivate them to make lifestyle changes.

## Discussion

This study explores the experiences of a sample of older adults who attended a lifestyle intervention programme with respect to their own lifestyle and being at increased risk for type 2 diabetes. The sample was selected from individuals who had participated at the HUNT3 Study and the HUNT DE-PLAN Study, and who were currently participating in the VEND-RISK Study. The first main theme that emerged was having resources available to live an active lifestyle, including having a family and being part of a social network, having a positive attitude to life, and maintaining healthy habits from childhood to the present. The second main theme that of being at increased type 2 diabetes risk, included varied reactions to the risk information, attending a lifestyle intervention programme (VEND-RISK) raised awareness about risk behaviour, and health-related worries and ambitions as type 2 diabetes prevention.

### Importance of resources and the ability to use them

In this study, the resources of having a family and being part of a social network were important in maintaining an active lifestyle among this selected sample of individuals at increased risk for type 2 diabetes. Another qualitative study on individuals in lifestyle programs showed that a negative experience with family and social life was debilitating and a barrier to making lifestyle changes [34]. Several studies have found that having a good social network is a protective factor against morbidity [35]. In addition, according to the salutogenic theory, social belonging is important for a positive health outcome [15]. People with partners, family and friends who provide psychological and material support have better health than people with poor social connections [36]. Furthermore, the motivational aspects of social relationships are associated with increased activity [37–39].

In our data, informants described themselves as active and having a positive attitude to life and considered themselves as persons who did not give up when they faced difficulties. These characteristics provided by our sample coincided with the salutogenic theory where individuals with a high sense of coherence have an ability to manage difficult situations alone or with the support of significant others [15]. To find meaning in a situation, for example through activities, is also an important part of the sense of coherence concept [14]. Thus, high sense of coherence affects an individual’s health positively over time in helping them handle stress and adapt to a healthier lifestyle [15]. Strengthening the sense of coherence for individuals in need, could play a role in trying to help them handle type 2 diabetes risk [17]. Focusing on and assessing sense of coherence may be useful in improving the outcome of lifestyle interventions [16]. Individuals with few available resources could be in need of more intensive follow-up from health personnel to help them make lifestyle changes than those with more resources.

### Perceived risk related to family history

Our results revealed different reactions to the risk information and differences in actions taken to live a healthier life. The informants described themselves as active, and continued with established lifestyle habits without making an effort to prevent type 2 diabetes. This is in accordance with one study that found risk perception among individuals with type 2 diabetes to be based on non-modifiable factors, suggesting they underestimated the impact of behavioural factors [40]. From a theoretical perspective, perception of risk itself is not sufficient to motivate an individual to make a change [41]. Nevertheless, increasing a perception of risk may result in some kinds of healthier lifestyles [21, 22, 42].

Individuals at risk with a family history of diabetes may worry more about developing the disease, compared to those without diabetes in the family [43]. Furthermore, those with the most negative secondary experiences with type 2 diabetes can construct excessive cognitive and emotional responses to the disease [43]. Studies have shown that when health care providers have information and knowledge about a person’s family history of diabetes, this can have a temporary effect on behavioural changes in individuals at risk of type 2 diabetes [44]. Other studies suggest that the usefulness of family history may however depend on the way information about risk is understood and perceived by those with increased risk [18, 45]. Nevertheless, in preventive strategies, a family history of diabetes may be of importance [46], and health personnel are open to using family history for such purposes [47]. In our study, a family history of diabetes was experienced more important than risk information in regard to participants’ perceiving a need for lifestyle change.

We recommend future intervention programmes to assess each individual perception of risk, with the goal of finding people who need more knowledge about the importance of modifiable factors in lifestyle change. Furthermore, even if health personnel initially focus on family history to initiate lifestyle change, an individual’s understanding of risk may be more important in helping with lifestyle improvements.

### Strengths and limitations

A qualitative design may provide insight to complex phenomena. Our selected sample of participants from HUNT DE-PLAN and VEND-RISK would have been difficult to recruit without using a nested design. Adding interviews to a quantitative study (VEND-RISK) adds independent knowledge about lifestyle change for individuals at increased risk for type 2 diabetes.

Using only one researcher to collect all data could be a limitation [48]. In the present study the first author conducted all interviews. In order to minimize potential biases, the co-authors (ASH and MS) read three interviews and a summary of all interviews. ASH and MS also analysed and discussed the data with ISF until they reached consensus on the results. The present study may be affected by recall bias since there was a gap of eight years between the information on risk that was provided at HUNT3 and participation in the VEND-RISK Study. Also, participants had attended two lifestyle intervention (HUNT DE-PLAN and VEND-RISK) studies before being interviewed. The HUNT DE-PLAN Study and the VEND-RISK Study had similar content, but differed in their locations and the intensity of their interventions.

Informants in the present study were selected because they were individuals at increased risk for type 2 diabetes and could be different from those from the HUNT DE-PLAN Study who chose not to participate when invited to the VEND-RISK Study. Also, they could differ from those who did not attend the HUNT DE-PLAN from HUNT3. We assume that the participants we included were engaged in improving health and avoiding type 2 diabetes. However, the aim of the study was to explore these engaged participants’ perceptions when attending a lifestyle intervention, as they also had been previous participants in a lifestyle intervention study. Other studies have found that people who choose not to participate in lifestyle intervention studies might be those who would have benefitted the most from the intervention [49]. In addition, the informants’ ages limit the transferability of the findings to all individuals at increased risk of type 2 diabetes who participate in lifestyle intervention programmes. However, these results are an important supplement to knowledge about older adults’ experience of lifestyle and increased type 2 diabetes risk.

## Conclusions

Having access to individual and social resources was shown by this study to be important for an active lifestyle. For this reason, helping individuals who need to strengthen their resources and the ability to use resources might be important in improving the outcomes of lifestyle intervention programmes. Concerns related to family history of diabetes and ambitions for a long and healthy life were more important in inducing lifestyle changes than information about risk. Our study indicates that improving an individual’s understanding of risk as a supplement to using family history in preventive strategies might be helpful in inducing lasting lifestyle changes. As there is a growing need to prevent type 2 diabetes, these results are important when planning and improving lifestyle interventions and health promotion programmes.

## Abbreviations

BMI: Body mass index
DE-PLAN: Diabetes in Europe prevention through lifestyle, physical activity and nutrition
FIND-RISC: The finnish diabetes risk score questionnaire
HUNT3: The Nord-Trøndelag health study 3
REK: The Regional Committee for Medical and Health Research Ethics in Central Norway
SD: Standard deviation
VEND-RISK: The lifestyle intervention programme in two municipalities in North-Trøndelag
